# Supplementary material for: Adaptation of the Protocol for the Isolation of Biotinylated Protein Complexes for Drosophila melanogaster Tissues
Source: Int J Mol Sci. 2025 Aug 19;26(16):8009. doi: 10.3390/ijms26168009 (PMC12386920; doi:10.3390/ijms26168009)
Supplement: Supplementary file 1 [file ijms-26-08009-s001.zip › Supplementary Methods.pdf]

# ADAPTATION OF THE PROTOCOL FOR THE ISOLATION OF BIOTINYLATED PROTEIN COMPLEXES FOR *DROSOPHILA MELANOGASTER* TISSUES.

**Shokodko I.A.[1]*(nero.fact@gmail.com), Ziganshin R.H.[2], Vorobyeva N.E. [1*](nvorobyova@gmail.com)**

[1] Institute of Gene Biology Russian Academy of Sciences, 119334, Moscow, info@genebiology.ru

[2] Shemyakin-Ovchinnikov Institute of Bioorganic Chemistry Russian Academy of Sciences, 117997, Moscow, office@ibch.ru

* To whom correspondence should be addressed

## SUPPLEMENTARY METHODS

**Biotin antibodies for the enrichment of biotinylated proteins.** Biotin antibodies (Abcam ab53494) were bound to Mab-select sepharose (GE Healthcare) in a 15V MN buffer (20 mM HEPES KOH pH 7.9; 100 mM KCl; 0.1% NP-40; 5 mM MgCl2) for an hour at room temperature. For one experiment, 20 µl of sepharose and 5 µl of biotin antibodies were used. Then, sepharose was washed with 20V BB buffer (0.3 M sodium borate pH 9; 0.3 M H3BO3; 60 mM NaOH) 3 times (each round for 3 minutes). The antibodies were immobilized on Mab-select Sepharose by using 5 mg of dimethyl pimelimidate (DMP, Sigma-Aldrich), dissolved in 1 ml of BB buffer. 20V freshly mixed BB+DMP was added to the sepharose with antibodies. Then, incubated for 30 minutes on the rotator at room temperature and centrifuged at 1000 *g* for 3 minutes (operation was performed 3 times) to remove the supernatant. After immobilization, sepharose was washed with 50V BB+Gly (BB buffer, 50 mM Gly) and incubated in this buffer on the rotator at 4°C for 12 hours. Then washed twice with RIPA buffer (50 mM Tris; 150 mM NaCl; 0.1% SDS; 0.5% sodium deoxycholate; 1% Triton X-100; pH 7.5) [1]. RIPA buffer was selected for these washes due to its stringent conditions, which are essential for efficient lysis and extraction of proteins from complex *Drosophila* tissue samples.

**Cell/tissue lysate protein precipitation.** S2 Shneider cells of *D.melanogaster* were centrifuged at 1000 *g* for 5 minutes at 4°C. After supernatant removal, 1 ml of MN buffer was added, then centrifuged at 1000 *g* for 5 minutes at 4°C. Cells were lysed in 500 µl of RIPA buffer + PIC (Roche) by sonication (2 rounds for 10 seconds).

*D.melanogaster* ovaries were collected manually in MN buffer, then stored at -70°C. Thawed tissues were homogenized by Douncer in the RIPA+PIC buffer. Then, the tissues were

lysed by sonication (3 rounds for 10 seconds) and treated with DNase I (ThermoScientific) for 10 minutes at 4°C. Upon centrifugation for 20 min at 4°C 21000 *g*, the finished lysate was precipitated by sepharose with biotin antibodies attached or by streptavidin-agarose (Sigma-Aldrich) using rotator at 4°C for 90 minutes. Then, sepharose was washed for 3 times with RIPA buffer. Precipitated proteins were eluted with SDS 2% elution buffer (2% SDS; 100 mM; TrisHCl pH 8.0; 0.5 mM EDTA) at 50°C/75°C/95°C for 5 minutes.

**Protease-resistant streptavidin sepharose (prS).** To produce protease-resistant streptavidin the previously described protocol was used [2]:

1. Add 1 volume (1V) of streptavidin-agarose to a microcentrifuge tube.
2. Centrifuge for 3 minutes at 3000 rpm and discard the supernatant.
3. Wash the sepharose with 2 volumes (2V) of PBS + 0.1% Tween.
4. Centrifuge for 3 minutes at 3000 rpm and discard the supernatant.
5. Resuspend the beads in 2.8V of **Reagent CHD**.
6. Incubate on a rotator at room temperature for 4 hours.
7. Centrifuge for 3 minutes at 3000 rpm and discard the supernatant.
8. Wash the sepharose with 2V of PBS + 0.1% Tween.
9. Centrifuge for 3 minutes at 3000 rpm and discard the supernatant.
10. Resuspend the sepharose in 1.5V of **Reagent A**.
11. Add 1.5V of **Reagent B**.
12. Incubate on a rotator at room temperature for 2 hours.
13. Centrifuge for 3 minutes at 3000 rpm and quickly discard the supernatant.
14. Wash the sepharose with 2V of 0.1 M Tris–HCl pH 7.5.
15. Centrifuge for 3 minutes at 3000 rpm and quickly discard the supernatant.
16. Wash the sepharose with 2V of PBS + 0.1% Tween.
17. Centrifuge for 3 minutes at 3000 rpm and quickly discard the supernatant.
18. Resuspend the sepharose in 1V of PBS + 0.1% Tween.
19. Store the modified sepharose at +4°C.

Reagents:

- **Reagent CHD**: Dissolve 360 mg of 1,2-cyclohexanedione in 42 ml of freshly prepared PBS + 0.1% Tween, adjusted to pH 13.
- **Reagent A** (toxic): 4% formaldehyde (v/v) in PBS + 0.1% Tween.
- **Reagent B** (toxic): 0.2 M sodium cyanoborohydride ($NaBH_3CN$) in PBS + 0.1% Tween.

**Transgenic *D.melanogaster*.** The coding region of the EcR-A isoform was fused with TurboID (TurboEcR) ligase (the latter obtained from Addgene plasmid # 107171), marked with a

triple FLAG epitope, and integrated into the attBdir-white-rev-pSK [3]. 10XUAS sites with minimal *hsp43* promoter were used to control the expression [4]. The created plasmid was integrated into *attP2* site using phiC31 system [5]. For the negative control in the experiment we used the transgenic fly stock bearing the same construct but lacking EcR-A coding region (TurboGly).

To achieve expression of TurboEcR and TurboGly fused proteins in ovarian somatic cells, the resulting transgenic flies were crossed with drivers carrying GAL4 under the control of *tj* promoter (AA274 stock previously obtained by A. Aravin). For ovarian tissue collection, recently eclosed females (no later than 16 hours after eclosion) were raised at the biotin-containing food (100 nM/ml concentration) for 1 day before the ovaries collection. Ovarian tissue dissection was performed manually. Collected tissues were stored at -70°C.

In total 120 ovaries for each TurboID-EcR and TurboID-Gly fly stocks were collected. For each stock lysate preparation and protein enrichment were performed in two completely separate biological replicates (60 ovary per one replica). The obtained tissue lysates were tested for the presense of transgenic proteins and biotinylated proteins. 1/5 of the total protein extract volume from each of the resulting replicates was pooled together, precipitated with prS, and subjected to Western blot analysis.

**Peptide fingerprinting and LC-MS analysis**. To compare quantitatively amount of the proteins precipitated from the tissues containing TurboEcR relative to control TurboGly tissues, lysates were prepared in parallel, precipitated with prS sepharose and washed by PBS before the trypsinolisys. Protein elution was performed by trypsinolysis followed by LC-MS/MS. Reduction, alkylation and digestion of the proteins were performed as described previously [6] with minor modifications. 20 μl of sodium deoxycholate (SDC) reduction and alkylation buffer pH 8.5 contained 100 mM TRIS, 1% (w/v) SDC, 10 mM TCEP and 20 mM 2-chloroacetamide were added to a protein sample. The sample was heated at 85°C for 10 min, cooled to a room temperature and 5 μl of trypsin solution (0.05 μg/μl) in 100 mM TRIS pH 8.5 was added. After overnight digestion at 37°C, peptides were acidified by 20 μl of 2% trifluoroacetic acid (TFA) mixed with 40 μl of ethyl acetate and loaded on SDB-RPS StageTips contained two 14-gauge SDB-RPS plugs, and the StageTip was centrifuged at 300 g until all solution go through the StageTip (typically 5 min). After washing the StageTips with a 100 μl of 1% TFA/ethyl acetate 1:1 mixture (2 times) and 50 μl of 0.2% TFA, peptides were eluted in a clean tube by 60 μl 50% acetonitrile/5% ammonia mixture using centrifugation at 300 g. The collected material was vacuum-dried and stored at -80°C. Before analyses peptides were dissolved in 15 μl of 2% acetonitrile/0.1% TFA buffer.

The data was processed by MaxQuant (https://www.maxquant.org). Subsequent data frames analysis, GeneOntology (GO) functional enrichment of the proteins (to determine their cell localization), and Volcano-plots graphing were performed using ad-hoc Python scripts with the Pandas, Numpy, Matplotlib, Plotly and adjustText packages.

## REFERENCES


1. Branon, T. C., Bosch, J. A., Sanchez, A. D., Udeshi, N. D., Svinkina, T., Carr, S. A., ... & Ting, A. Y. (2018). Efficient proximity labeling in living cells and organisms with TurboID. *Nature biotechnology*, *36*(9), 880-887, doi: 10.1038/nbt.4201.
2. Rafiee, M. R., Sigismondo, G., Kalxdorf, M., Förster, L., Brügger, B., Béthune, J., & Krijgsveld, J. (2020). Protease-resistant streptavidin for interaction proteomics. *Molecular systems biology*, *16*(5), e9370, doi: 10.15252/msb.20199370.
3. Erokhin, M., Gorbenko, F., Lomaev, D., Mazina, M. Y., Mikhailova, A., Garaev, A. K., ... & Chetverina, D. (2021). Boundaries potentiate polycomb response element-mediated silencing. *BMC biology*, *19*(1), 113, doi: 10.1186/s12915-021-01047-8.
4. Vorobyeva, N. E., Soshnikova, N. V., Nikolenko, J. V., Kuzmina, J. L., Nabirochkina, E. N., Georgieva, S. G., & Shidlovskii, Y. V. (2009). Transcription coactivator SAYP combines chromatin remodeler Brahma and transcription initiation factor TFIID into a single supercomplex. *Proceedings of the National Academy of Sciences*, *106*(27), 11049-11054, doi: 10.1073/pnas.0901801106.
5. Markstein, M., Pitsouli, C., Villalta, C., Celniker, S. E., & Perrimon, N. (2008). Exploiting position effects and the gypsy retrovirus insulator to engineer precisely expressed transgenes. *Nature genetics*, *40*(4), 476-483, doi: 10.1038/ng.101.
6. Kulak, N. A., Pichler, G., Paron, I., Nagaraj, N., & Mann, M. (2014). Minimal, encapsulated proteomic-sample processing applied to copy-number estimation in eukaryotic cells. *Nature methods*, *11*(3), 319-324, doi: 10.1038/nmeth.2834.
