# Supplementary material for: Adaptation of the Protocol for the Isolation of Biotinylated Protein Complexes for Drosophila melanogaster Tissues
Source: Int J Mol Sci. 2025 Aug 19;26(16):8009. doi: 10.3390/ijms26168009 (PMC12386920; doi:10.3390/ijms26168009)
Supplement: Supplementary file 1 [file ijms-26-08009-s001.zip › WesternsSupplementary.pdf]

ADAPTATION OF THE PROTOCOL FOR THE ISOLATION OF BIOTINYLATED PROTEIN COMPLEXES FOR *DROSOPHILA MELANOGASTER* TISSUES.

Shokodko I.A. 1(nero.fact@gmail.com),
Ziganshin R.H.2,
Vorobyeva N.E. 1*(nvorobyova@gmail.com)

1 Institute of Gene Biology Russian Academy of Sciences, 119334, Moscow, info@genebiology.ru
2 Shemyakin-Ovchinnikov Institute of Bioorganic Chemistry Russian Academy of Sciences, 117997, Moscow, office@ibch.ru
* To whom correspondence should be addressed

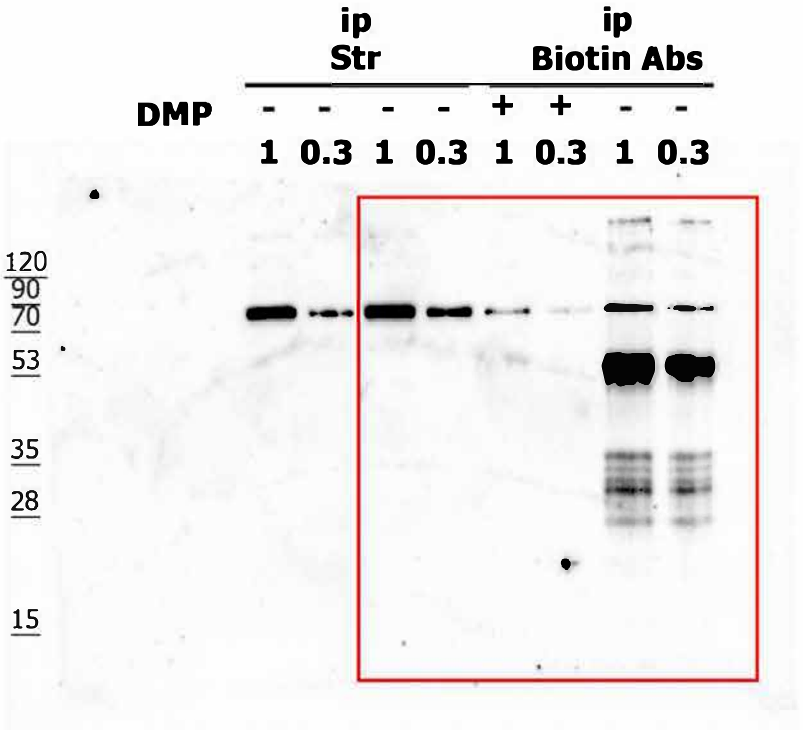

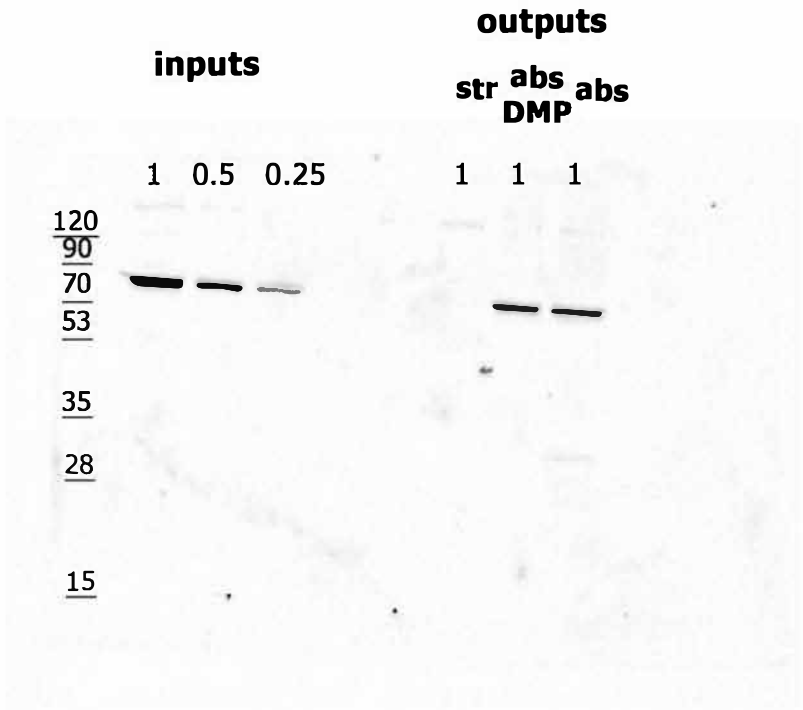

Supplementary figure S1. The original Western blots (full-size) from the Fig. 1

A

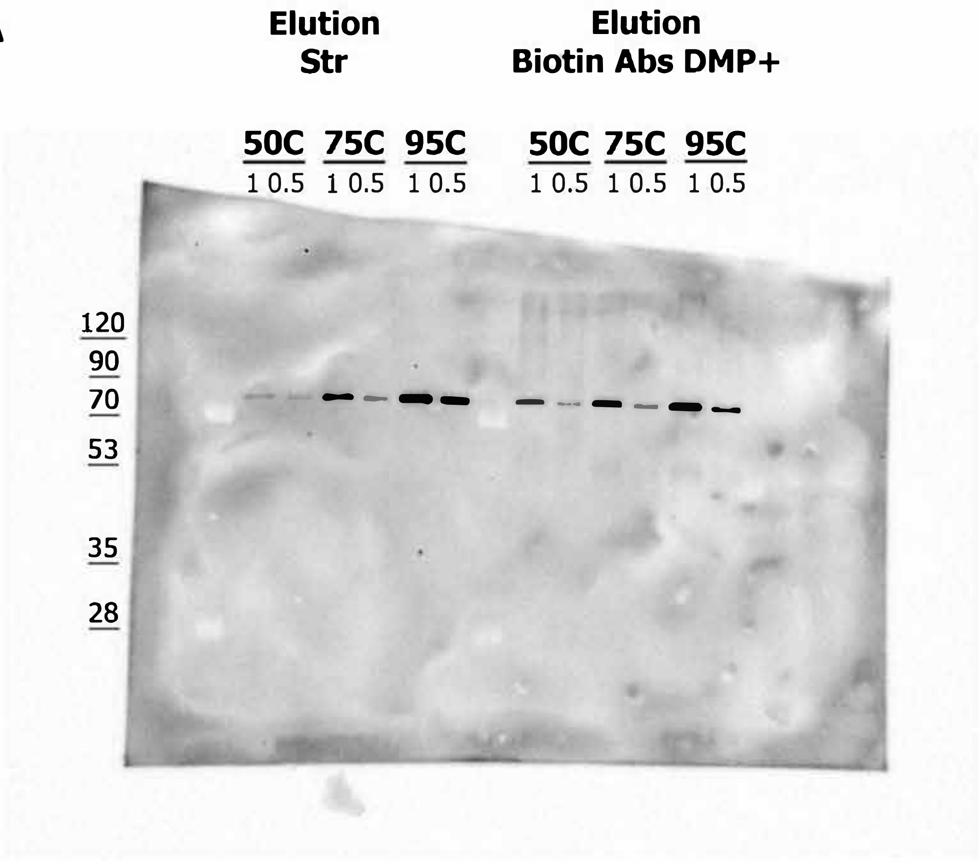

B

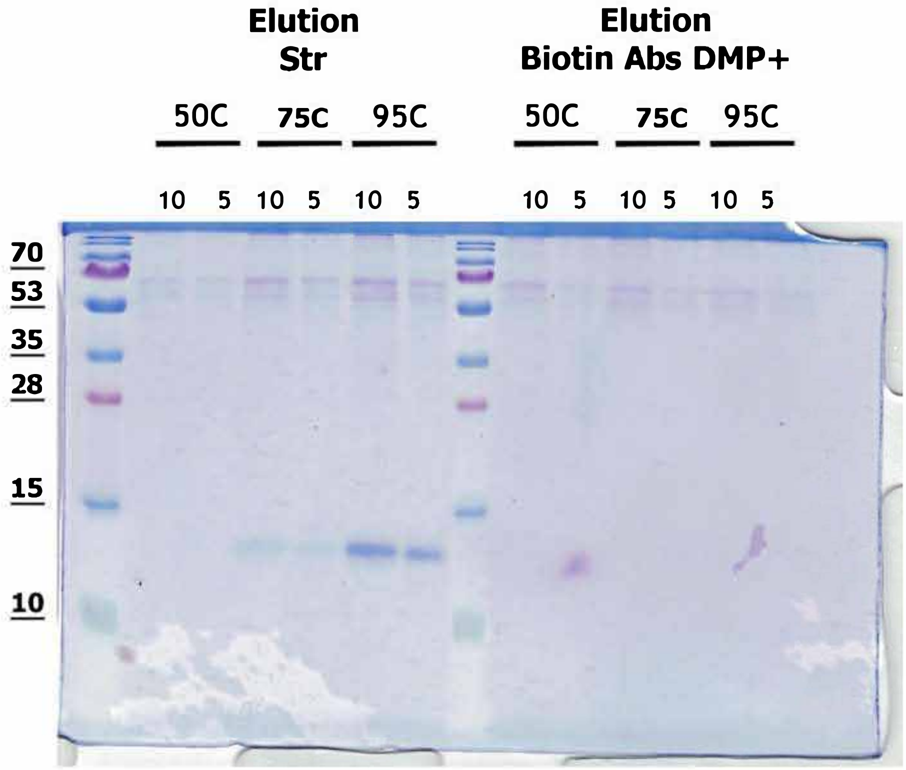

Supplementary Figure S2. The original Western blots (full-size) from the Fig. 2

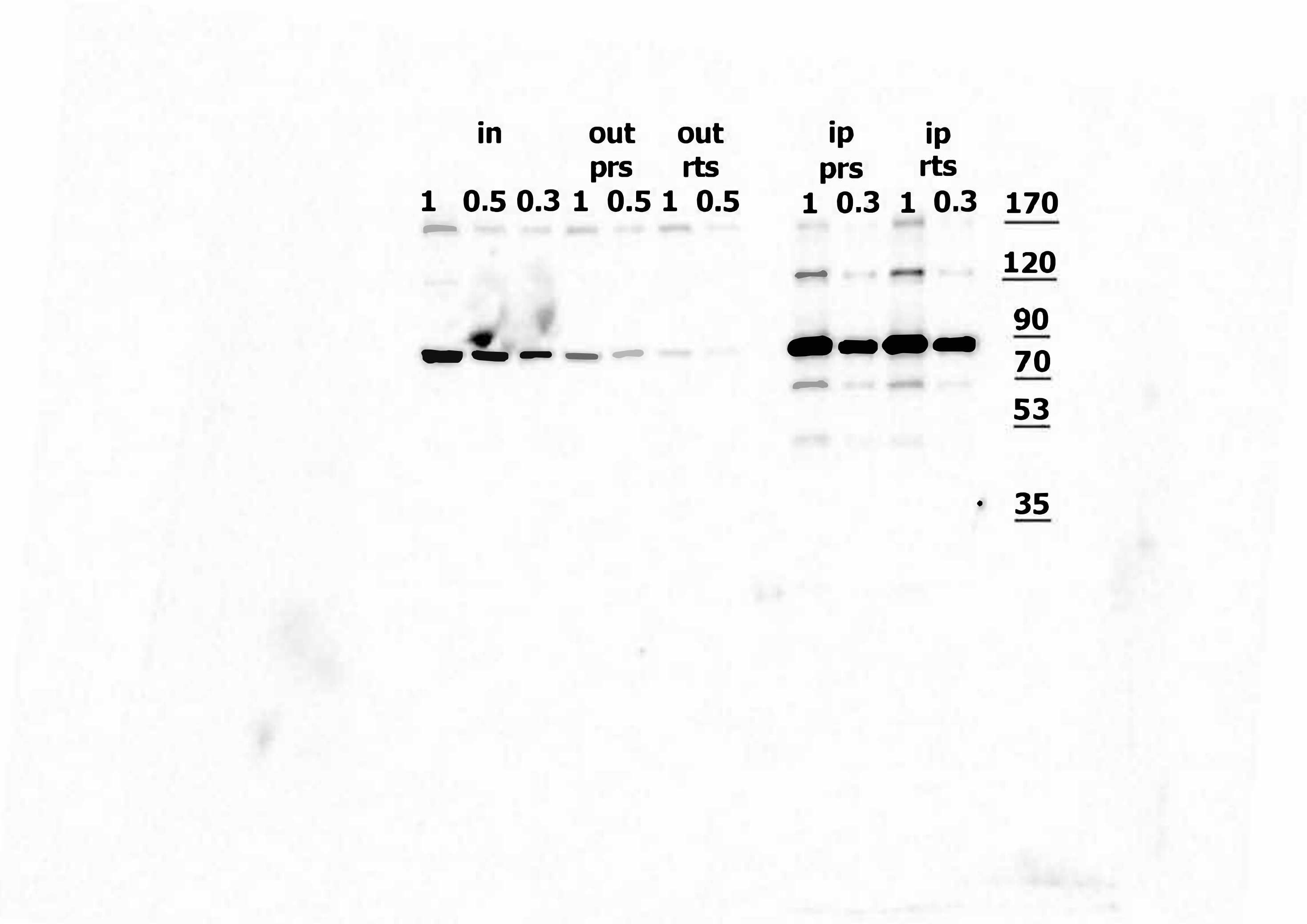

Supplementary figure S3. The original Western blots (full-size) from the Fig. 3

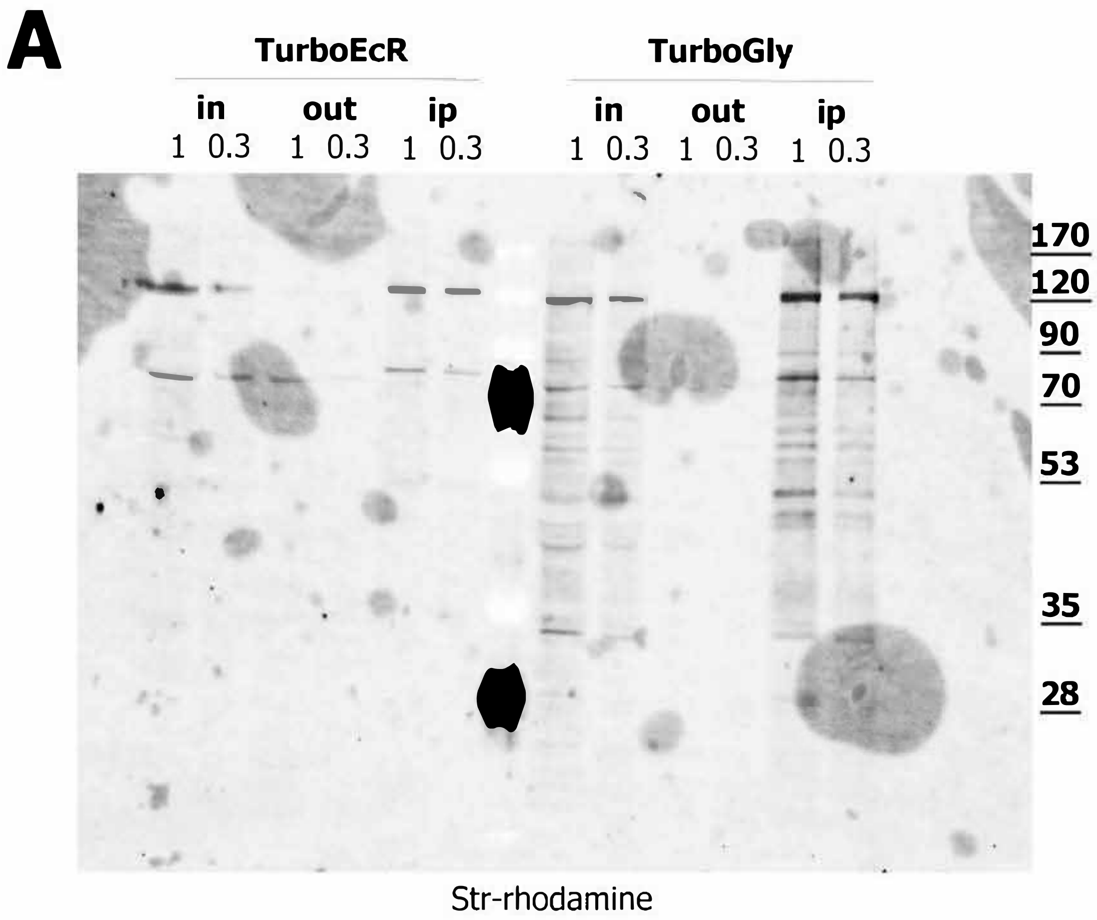

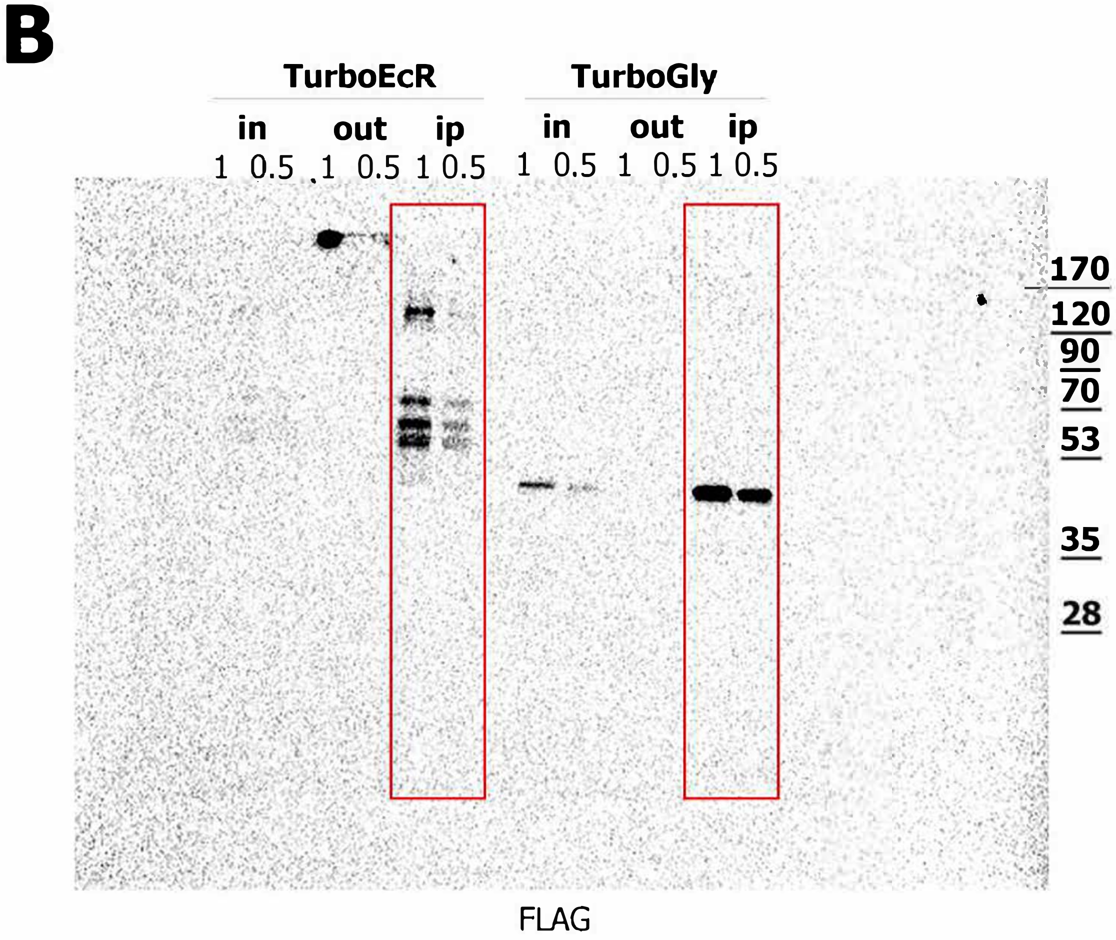

Supplementary figure S4. The original Western blots (full-size) from the Fig. 6
